# A Rod-like Bi_2_O_3_ Photocatalyst Derived from Bi-Based MOFs for the Efficient Adsorption and Catalytic Reduction of Cr(VI)

**DOI:** 10.3390/ijms252313052

**Published:** 2024-12-04

**Authors:** Qin Fang, Luying Chen, Qiucheng Fu, Yongjuan Chen, Jiao He, Liang Jiang, Zhiying Yan, Jiaqiang Wang

**Affiliations:** 1 School of Chemical Sciences & Technology, Yunnan University, Kunming 650091, China; fangqin1017@163.com (Q.F.); cly159365128122022@163.com (L.C.); 12022116104@mail.ynu.edu.cn (Q.F.); yongjuanchen@ynu.edu.cn (Y.C.); hejiao@ynu.edu.cn (J.H.); 2 Yunnan Province Engineering Research Center of Photocatalytic Treatment of Industrial Wastewater, Yunnan University, Kunming 650091, China; jiangliang@ynu.edu.cn; 3 Engineering Institute, Yunnan University, Kunming 650091, China; 4 School of Materials & Energy, Yunnan University, Kunming 650091, China

**Keywords:** MOF-derived Bi_2_O_3_, Bi-BTC, Cr(VI), photocatalysis

## Abstract

Heavy metal ion pollution poses a serious threat to the natural environment and human health. Photoreduction through Bi-based photocatalysts is regarded as an advanced green technology for solving environmental problems. However, their photocatalytic activity is limited by the rapid recombination of photogenerated e^−^ and h^+^ pairs and a low photo-quantum efficiency. In this work, an optimal precursor of Bi-based MOFs was identified by using different solvents, and rod-like Bi_2_O_3_ materials were derived by in situ oxidation of Bi atoms in the precursor. The adsorption and photocatalytic reduction efficiency of the prepared Bi_2_O_3_ materials for Cr(VI) were evaluated under visible light irradiation. The results showed that the prepared materials had a large specific surface area and enhanced visible light absorption. Bi_2_O_3_(DMF/MeOH-3)-400 had a large specific surface area and many active adsorption sites, and it had the highest adsorption of Cr(VI) (49.13%) among the materials. Bi_2_O_3_(DMF/MeOH-3)-400 also had the highest photocatalytic reduction efficiency, and it achieved 100% removal of 10 mg·L^−1^ Cr(VI) within 90 min under light. In addition, the material showed remarkable stability after three consecutive photocatalytic cycles. The enhanced photocatalytic performance was mainly attributed to the fast separation of electron–hole pairs and efficient electron transfer in the MOF-derived materials, which was confirmed by electrochemical tests and PL spectroscopy. Reactive species trapping experiments confirmed that electrons were the main active substances; accordingly, a possible photocatalytic mechanism was proposed. In conclusion, this work provides a new perspective for designing novel photocatalysts that can facilitate the removal of Cr(VI) from water.

## 1. Introduction

Chromium has a variety of applications in industry, and the improper disposal of chromium-containing industrial waste can cause serious environmental pollution. Chromium exists in nature mainly in the form of Cr(VI) and Cr(III) and the metallic form Cr(0); Cr(VI), mainly in the form of HCrO^4−^ or CrO_4_^2−^, is more hazardous due to its high water solubility and strong carcinogenic effect on most organisms. Therefore, exploring appropriate methods for removing Cr(VI) is crucial. Various methods, including adsorption [1], chemical precipitation, membrane filtration, and reduction from highly toxic Cr(VI) to less toxic Cr(III) [2], have been reported for Cr(VI) wastewater treatment. One of the most preferred methods of effective remediation of Cr(VI)-containing wastewater is the transformation of Cr(VI) to Cr(III), because Cr(III) has low toxicity and can be easily precipitated and removed as a solid waste, such as in the form of Cr(OH)_3_ [3]. Compared with chemical reduction, photocatalytic reduction of Cr(VI) to Cr(III) using photocatalysts is more effective and of lower cost and does not produce hazardous chemicals [3]. Like any other semiconductor, bismuth-based bismuth oxide (Bi_2_O_3_) as a semiconductor material has attracted considerable attention because of its good dielectric, optical, and ionic conductivity properties and its biocompatibility and environmental friendliness [4]. However, the photocatalytic activity of Bi_2_O_3_ is limited by the rapid recombination of photogenerated e^−^ and h^+^ pairs and its low photo-quantum efficiency [5].

To solve this problem, researchers have attempted various methods, including morphological modification, ion or nonmetallic element doping, and heterogeneous structure building. For example, Wu et al. prepared various morphological bismuth oxides (α-Bi_2_O_3_) by a self-assembly process using bismuth oxalates as precursors. They found that a honeycomb brush-like Bi_2_O_3_ showed superior photocatalytic degradation of acid orange 7 dye [6]. Abdul et al. added an appropriate amount of bismuth oxide formate to bismuth citrate and calcined it in air at 350 °C to obtain nanoparticle-based multilayered flakes of β-Bi_2_O_3_, which showed a photodegradation rate of 92.9% for TC under visible light irradiation [7]. Charu et al. synthesized a novel nanocomposite Bi_2_O_3_/Sb_2_S_3_ via an environmentally friendly hydrothermal pathway and photocatalytically decomposed RhB and TC under sunlight irradiation to achieve efficient degradation of dyes and antibiotics [8]. Although these studies have made some progress, there are still challenges and issues that need to be solved, including the appropriate synthetic methods and optimization of catalysts.

One facile method for Bi_2_O_3_ material preparation is solid-state thermal decomposition of meta-organic precursors at suitable temperatures. In previous reports, most researchers adopted traditional, simple metal-oxalate complexes as precursors. Recently, metal-organic framework (MOF) derivatives, including porous carbon, metal oxides, and metal sulfides, were used in adsorption, catalysis, and energy storage and conversion [9,10]. They retain the merits of their MOF precursors, such as a high specific surface area, abundant pores, and a controllable morphology. Furthermore, the derived compounds have higher electrical properties and greater stability than MOFs themselves. These special properties and structures can reduce recombination of photogenerated electron–hole pairs and improve their visible light absorption, thus improving their photocatalytic activity [11]. Therefore, the rational use of MOFs to derive photocatalysts with excellent photocatalytic activity has attracted great attention. For example, Song et al. prepared visible-light photocatalysts of Bi_2_O_3_/Bi embedded in porous carbon from Bi-based MOFs for highly efficient rhodamine B removal from water [12]. Guo et al. generated MOF-derived Bi_2_O_3_@C with rich oxygen vacancies through rapid thermal annealing technology. The removal rate of tetracycline hydrochloride reached 88% after 120 min of irradiation [13]. Although current Bi-based MOF derivatives can efficiently improve the performance of photocatalysts, there are few reports on the preparation of single-phase Bi_2_O_3_ and its application in the photocatalytic reduction of heavy metals [14]. More importantly, the morphology and structure of Bi-based MOF derivatives largely depend on the solvent and the type of ligand used to prepare MOFs [14]. In addition, the calcination temperature also has a great influence on the structure of MOF derivatives. Therefore, to obtain photocatalysts with high performance, how to optimize the preparation process is still a problem that needs further research and exploration.

**Scheme 1:**
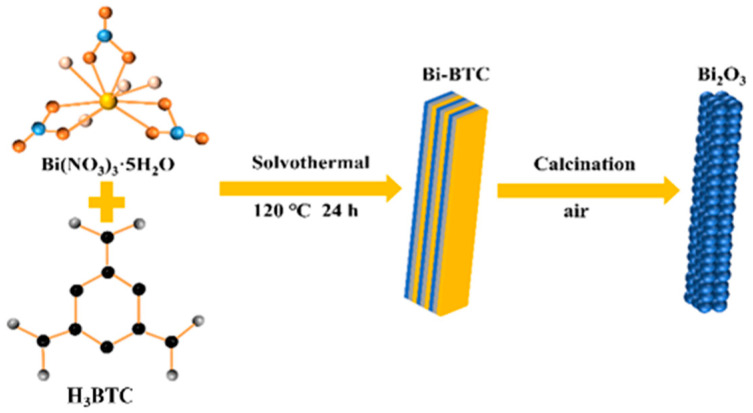
Synthetic route of the rod−like Bi_2_O_3_ samples.

Herein, we used Bi-BTC, a type of Bi-based MOF prepared using Trimesic Acid (H_3_BTC) as a ligand, to obtain a series of Bi_2_O_3_ photocatalysts under different calcination temperatures, as shown in Scheme 1. During the preparation, the optimal precursor Bi-BTC was screened by changing the solvent to N,N-Dimethylformamide (DMF), Methanol (MeOH), and a mixture of DMF and MeOH. It was found that the obtained Bi_2_O_3_ inherited the microscopic morphology of the precursor and formed a rod-like pore structure with closely arranged Bi_2_O_3_ nanoparticles. These features were beneficial to the adsorption of contaminants in water and improved the photocatalytic activity of the catalyst. The photocatalytic performance of the materials in the reduction of Cr(VI) under visible light was evaluated. As expected, the rod-like Bi_2_O_3_ from Bi-BTC, prepared with a mixed solvent of DMF and MeOH, showed excellent photocatalytic activity. Furthermore, the enhanced photocatalytic performance was confirmed by electrochemical tests and photoluminescence spectroscopy. In addition, experiments were performed to elucidate the mechanisms of the enhanced photocatalytic activity.

## 2. Results and Discussion

### 2.1. Characterization

**Figure 1:**
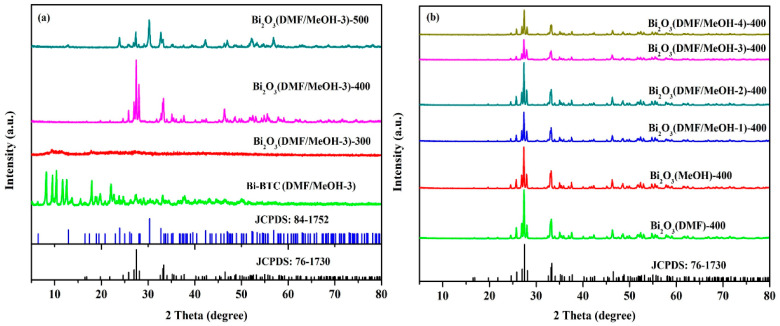
XRD patterns of Bi-BTC precursors and the as-prepared samples at 300 °C, 400 °C, and 500 °C (**a**), and using different solvents (**b**).

Information on the crystal structure of the materials was obtained using X-ray diffraction (XRD) analysis. As shown in Figure 1a, all diffraction peaks of the Bi-BTC precursors synthesized by using the mixed solvent DMF and MeOH were consistent with those of references, and there were no other peaks, indicating that the Bi-BTC precursor was successfully prepared [15]. When Bi-BTC (DMF/MeOH-3) was calcined at 300 °C in an air atmosphere for 2 h, the strong and sharp peaks disappeared, suggesting a change in the crystallinity and crystal structure of Bi-BTC, although new crystals were not generated [16]. When the calcination temperature increased to 400 °C, α-Bi_2_O_3_ with higher crystallinity was generated, and the diffraction peaks (2θ) at 27.5°, 33.2°, and 33.3° corresponded to the (1 2 0), (−1 2 1), and (2 0 0) crystal planes of Bi_2_O_3_ (JCPDS: 76-1730), respectively [17]. Further increasing the temperature to 500 °C, a new substance, Bi_2_O_2_CO_3_ (JCPDS: 84-1752), was generated; this may have been due to the reaction of Bi_2_O_3_ with the residual organic ligands in Bi-BTC at relatively high temperatures [18]. Accordingly, the calcination temperature of 400 °C was chosen to prepare Bi_2_O_3_ materials. Six different solvent/solvent ratios were chosen to synthesize the Bi-BTC precursors, which were heat-treated at 400 °C under an air atmosphere in a muffle furnace to obtain the materials shown in Figure 1b, all of which corresponded to α-Bi_2_O_3_ (JCPDS: 76-1730).

**Figure 2:**
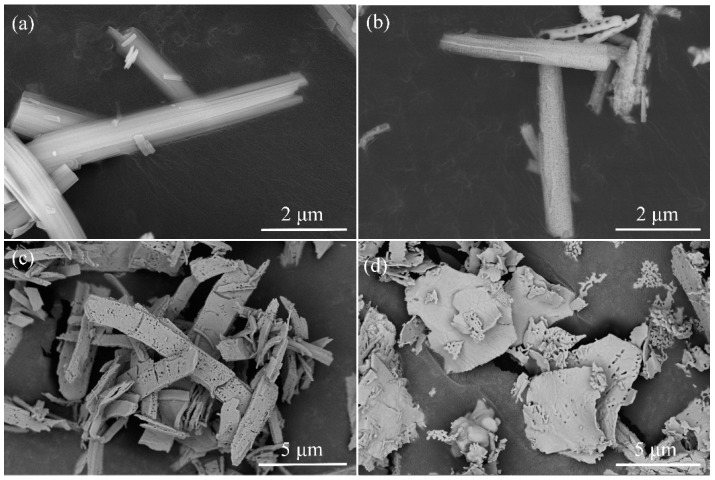
SEM images: (**a**) Bi-BTC(DMF/MeOH-3), (**b**) Bi_2_O_3_(DMF/MeOH-3)-400, (**c**) Bi_2_O_3_(DMF)-400, and (**d**) Bi_2_O_3_(MeOH)-400.

Figure 2 shows the scanning electron microscopy (SEM) images of the Bi-BTC precursor and the as-prepared samples. As shown in Figure 2a, the Bi-BTC precursor synthesized using a mixed solvent exhibited a relatively regular rod-like structure with a smooth surface. As shown in the SEM image in Figure 2b, Bi_2_O_3_(DMF/MeOH-3)-400 also exhibited the micrometer rod-like structure of the precursor Bi-BTC, but its surface became rough with a loose pore structure. The rods were about 6 μm in length and 0.7 μm in diameter. When the solvent was changed to DMF to synthesize Bi-BTC with further derivatization to Bi_2_O_3_, a thin slatted Bi_2_O_3_ material with a length of about 10 μm and a width of about 1 μm was obtained, with a clearly evident pore structure on the surface of the sample (Figure 2c). When MeOH was used as the solvent to prepare Bi-BTC, the derived Bi_2_O_3_ material consisted of irregular micron-scale sheets with a rough, fluffy surface and a few holes, as shown in Figure 2d. As shown in the SEM images, the synthesis of Bi-BTC using different solvents led to materials with different morphologies. When calcined in a muffle furnace, the obtained Bi_2_O_3_ inherited the morphological features of the original Bi-BTC, and all the materials had a porous structure, which can better adsorb hexavalent chromium, improve the efficiency of the separation and transfer of electrons and holes, and enhance the ability of the reduction of hexavalent chromium. However, due to high-temperature calcination, the structures of the materials were slightly fractured with some fragments.

**Figure 3:**
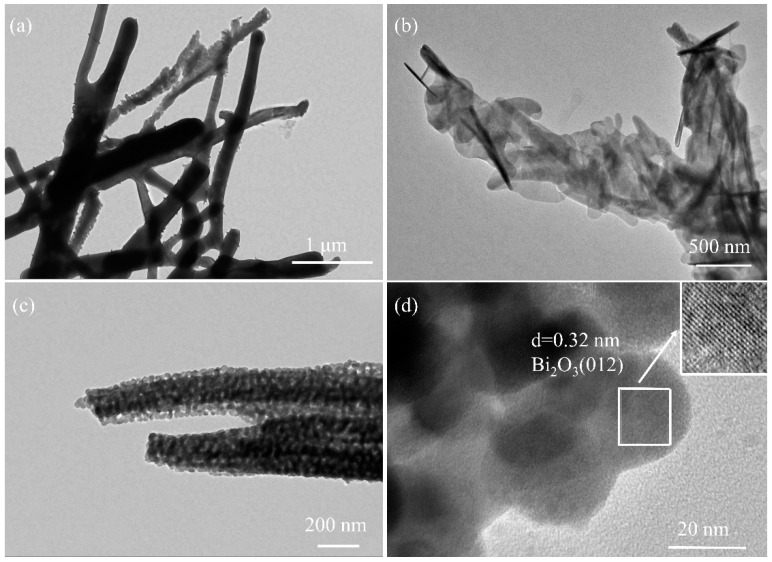
(**a**–**c**) TEM images of Bi_2_O_3_(DMF/MeOH-3)-400, and (**d**) HRTEM image of Bi_2_O_3_ (DMF/MeOH-3)-400.

Figure 3a–c show the transmission electron microscopy (TEM) images of the prepared Bi_2_O_3_(DMF/MeOH-3)-400 material, demonstrating that the structure of the precursor did not collapse under high-temperature calcination. This confirmed that the MOF derivatives had strong structural stability. As shown in Figure 3c, the metal Bi in the Bi-BTC skeleton was oxidized in situ to form Bi_2_O_3_ nanoparticles, which were tightly arranged into micrometer rod-like structures. This is the reason that the Bi_2_O_3_ remained in the same shape as the precursor. This multistage porous structure had fully exposed active sites, promoting the rapid transfer of photogenerated electrons and improving the separation and transfer efficiency of e^−^–h^+^. The HRTEM image of Bi_2_O_3_(DMF/MeOH-3)-400 (Figure 3d) indicated a facet spacing of 0.32 nm, which corresponded to the (012) facet of Bi_2_O_3_ [16].

**Figure 4:**
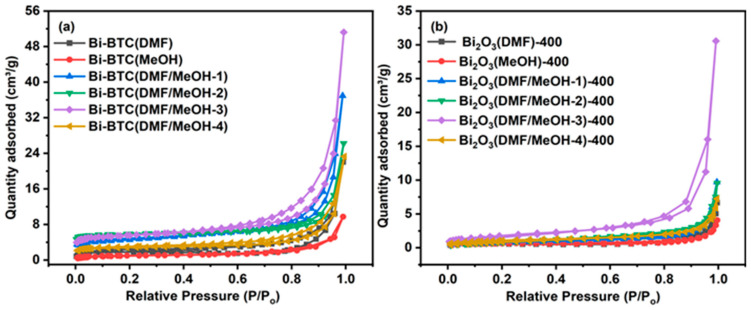
N_2_ adsorption–desorption isotherms were used to obtain the specific surface area and pore structure of Bi-BTC (**a**) and the as-prepared samples (**b**).

The N_2_ adsorption–desorption isotherm, as shown in Figure 4, was analyzed to obtain the specific surface area and pore structure of the samples. As shown in Figure 4a, the curves of the prepared Bi-BTC materials in the relative pressure range of 0.4–1 exhibited typical type IV isotherms and H3 hysteresis loops, confirming the coexistence of microporous and mesoporous structures [19]. The nitrogen adsorption–desorption isotherms of the Bi-BTC-derived Bi_2_O_3_ material shown in Figure 4b were similar to that of Bi-BTC, indicating that the derived Bi_2_O_3_ material inherited the pore structure of both micropores and mesopores. Table S1 presents the specific surface area and pore structure data of the prepared materials. The specific surface area of Bi-BTC prepared using the solvents DMF or MeOH alone was smaller than that of Bi-BTC prepared using mixed solvents, and Bi-BTC prepared using the mixed solvents DMF and MeOH (3:1) had the largest specific surface area (17.93 m^2^/g). This pattern was also present in the Bi-BTC-derived Bi_2_O_3_ material, with Bi_2_O_3_(DMF/MeOH-3)-400 exhibiting the largest specific surface area (6.60 m^2^/g). This result stemmed from the porous layered structure of the precursor, which provided more active sites for the reaction and was favorable for the photocatalytic reaction.

**Figure 5:**
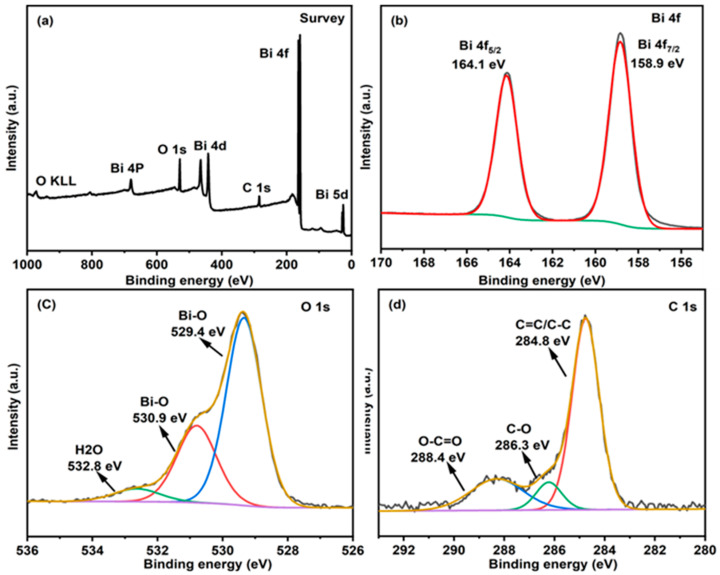
XPS spectra of Bi_2_O_3_(DMF/MeOH-3)-400: (**a**) survey scan, (**b**) Bi 4f, (**c**) O 1s, and (**d**) C 1s.

The surface elemental composition and chemical state of the Bi_2_O_3_ materials were analyzed using X-ray photoelectron spectroscopy (XPS). Figure 5a shows the full XPS spectrum of Bi_2_O_3_(DMF/MeOH-3)-400 with the presence of Bi, O, and C elements in the material, which further demonstrated the successful preparation of Bi_2_O_3_ with high purity. Figure 5b shows a high-resolution Bi 4f spectrum of Bi_2_O_3_(DMF/MeOH-3)-400 with two distinct peaks at binding energies of 158.9 eV and 164.1 eV, attributed to Bi 4f_7/2_ and Bi4f_5/2_, respectively. Figure 5c shows the high-resolution O 1s spectrum, with peaks at 529.4, 530.9, and 532.8 eV, corresponding to the Bi-O bonding in Bi_2_O_3_ and the adsorbed water on the surface [20,21,22]. As shown in Figure 5d, the C 1s spectrum of Bi_2_O_3_(DMF/MeOH-3)-400 was divided into three subpeaks at 284.8, 286.3, and 288.4 eV. The C 1s peak centered at 284.8 eV was associated with C=C/C-C [23], while the other two peaks at 286.3 eV and 288.4 eV were attributed to C-O and O-C=O, respectively [24,25]. This result suggested that Bi-BTC-derived Bi_2_O_3_ materials contained a certain amount of carbon, which was derived from the residual ligands in Bi-BTC, and that carbon improved the conductivity and promoted charge transfer, thus enhancing the photocatalytic efficiency of Bi_2_O_3_.

**Figure 6:**
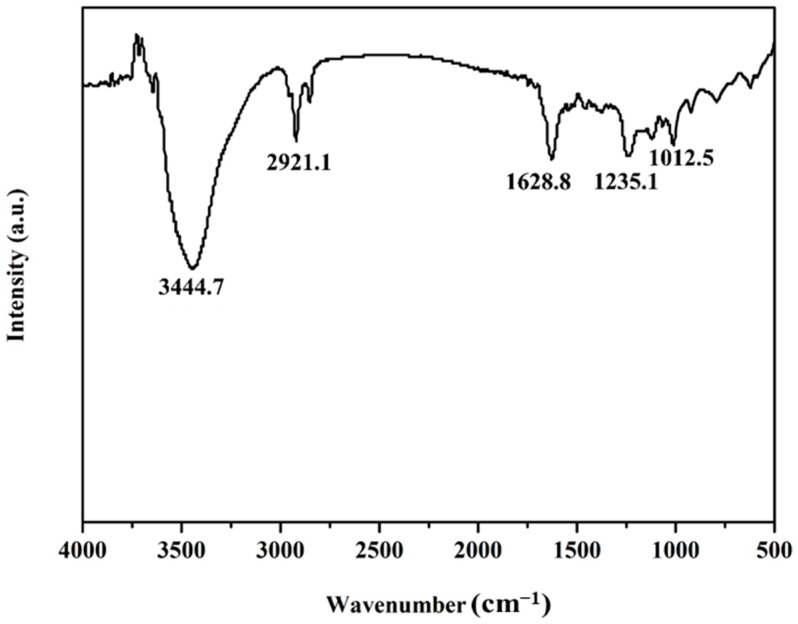
FT−IR spectrum of the Bi_2_O_3_(DMF/MeOH-3)-400 samples.

The Fourier transform infrared (FT-IR) spectra were analyzed to obtain information on the surface functional groups of the Bi_2_O_3_(DMF/MeOH-3)-400 material. As shown in Figure 6, the characteristic peak at 547.5 cm^−1^ was assigned to the stretching vibration of the Bi-O bond, and the peak at 845.8 cm^−1^ was attributed to the stretching vibration of Bi-O-Bi [26]. The broader absorption band at 3447.3 cm^−1^ was attributed to the stretching vibration of O-H in adsorbed water on the surface of the material, and the more pronounced characteristic peak at 1390.5 cm^−1^ corresponded to the stretching vibration of the aromatic C=C bond, which indicated the residual organic ligands in Bi-BTC [27].

**Figure 7:**
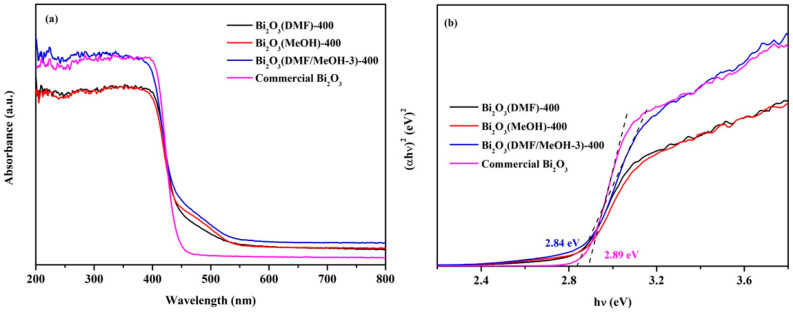
(**a**) UV−Vis DRS spectra and (**b**) Tauc plots of as-prepared samples.

The light absorption properties of the Bi-BTC-derived Bi_2_O_3_ materials and the commercial Bi_2_O_3_ sample were analyzed by UV–vis diffuse reflection spectra (UV–Vis DRS). As shown in Figure 7a, the maximum absorption wavelength of the Bi_2_O_3_ material was about 420 nm in the wavelength range of 200–800 nm, which indicated that it exhibited a good response to visible light. The absorption intensities of the Bi-BTC-derived Bi_2_O_3_ materials exhibited different enhancement between 420 and 800 nm compared with that of commercial Bi_2_O_3_, with the largest absorption intensity of Bi-BTC-derived Bi_2_O_3_ prepared by mixing with the solvent DMF/MeOH (3:1). Bi_2_O_3_(DMF/MeOH-3)-400 had the greatest absorption intensity, which may be related to the structure of the material itself. The band edges estimated by the Kubelka–Munk equation (Table S2) and corresponding Tauc plots (Figure 7b) clearly showed that the band gaps of Bi-BTC-derived Bi_2_O_3_ (2.84–2.88 eV) were lower than that of commercial Bi_2_O_3_ (2.89 eV) [28].

### 2.2. Photocatalytic Reduction of Cr(VI)

**Figure 8:**
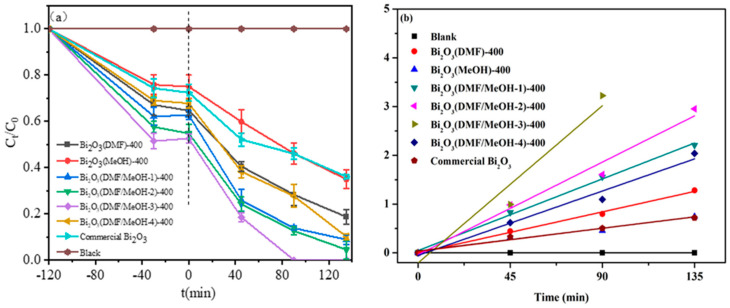
Photocatalytic reduction performance for Cr(VI) by the as-prepared samples and commercial Bi_2_O_3_ (**a**). The corresponding apparent first−order reaction kinetics curve (**b**).

The photocatalytic reaction was preceded by a full dark reaction, and all systems reached adsorption–desorption equilibrium after 90 min of the dark reaction. As shown in Figure 8a, at a pH of 2.5, the adsorption performance of the Bi-BTC-derived Bi_2_O_3_ materials synthesized using different solvents for Cr(VI) adsorption varied, but all the materials showed better adsorption performance than commercial Bi_2_O_3_. Bi_2_O_3_(DMF/MeOH-3)-400 had the greatest adsorption of Cr(VI) (49.13%), which may be attributed to the different morphologies of the original Bi-BTC when synthesized using different solvents. Furthermore, the Bi_2_O_3_ material inherited the microscopic pore structure of Bi-BTC, which led to a difference in specific surface area. Bi_2_O_3_(DMF/MeOH-3)-400 exhibited a larger specific surface area and more active adsorption sites. The concentration of Cr(VI) did not change after 135 min of visible light irradiation in the absence of a catalytic agent, indicating that Cr(VI) maintained a certain degree of stability and was not spontaneously degraded. The photocatalytic removal of hexavalent chromium by adsorption of the commercial Bi_2_O_3_ was poor, removing only about 64.95%. The Bi-BTC-derived Bi_2_O_3_ materials exhibited differing photocatalytic reductions of Cr(VI), and the synthesis of the Bi-BTC-derived Bi_2_O_3_ materials using DMF alone or MeOH as a solvent slightly outperformed that of commercial Bi_2_O_3_. The adsorptive and photocatalytic removal of Cr(VI) from the Bi-BTC-derived Bi_2_O_3_ materials synthesized using mixed solvents (DMF/MeOH) was significantly enhanced, with the best photocatalytic effect demonstrated by Bi_2_O_3_(DMF/MeOH-3)-400, which achieved 100% removal of 10 mg·L^−1^ Cr(VI) 90 min after light-on. As shown in Figure 8b, the kinetic curve of the photocatalytic reduction of Cr(VI) followed first-order kinetics [29]:

ln(C_t1_/C_t2_) = kt (1)

where C_t1_ is the concentration of Cr(VI) at the equilibrium of the dark reaction, and C_t2_ is the concentration of Cr(VI) after time t of light-on (min).

The apparent rate constants of the prepared samples and commercial Bi_2_O_3_ are listed in Table S3. The photocatalytic reduction efficiency of Cr(VI) of the Bi_2_O_3_ samples prepared by synthesizing Bi-BTC using a mixed solvent of DMF/MeOH was higher than that of the Bi_2_O_3_ samples prepared using solvent alone and that of commercial Bi_2_O_3_. Bi_2_O_3_(DMF/MeOH-3)-400 had the largest apparent rate constant (k = 0.035 min^−1^), which was seven times higher than that of commercial.

**Figure 9:**
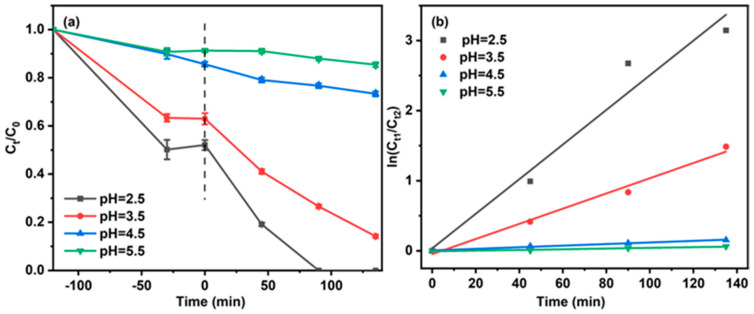
Performance of the photocatalyst in the reduction of Cr(VI) at different initial pH values (**a**). The corresponding apparent first−order reaction kinetics curve (**b**).

The effect of the initial solution pH on the adsorptive photocatalytic removal of Cr(VI) by Bi_2_O_3_(DMF/MeOH-3)-400 was investigated. As shown in Figure 9a, samples were exposed to a solution of 10 ppm Cr(VI) at different pH values, and the removal of Cr(VI) after 135 min of visible light irradiation by Bi_2_O_3_(DMF/MeOH-3)-400 was 100%, 85.89%, 26.69%, and 14.96% for an initial pH of 2.5, 3.5, 4.5, and 5.5, respectively. This indicated that an acidic environment was favorable for the adsorption and photocatalytic removal of Cr(VI) by Bi_2_O_3_ materials, and the adsorption and photocatalytic performances decreased dramatically when the pH value was close to neutral. Table S4 lists the apparent rate constants corresponding to different initial pH values of the Cr(VI) solution, with the largest apparent rate constant at pH 2.5 (0.035 min^−1^). This was attributed to the positively charged surface protonation of the Bi_2_O_3_ material under acidic conditions and the electrostatic attraction with HCr_2_O_7_^−^ and Cr_2_O_7_^2−^, which facilitated adsorption and the photocatalytic reaction [30]. Figure S1 shows the change in the zeta potential of Bi_2_O_3_(DMF/MeOH-3)-400 as the solution pH changed. The isoelectric point (pH_PZC_) of Bi_2_O_3_(DMF/MeOH-3)-400 was 4.9, and therefore, the surface of the material was positively charged at pH 2.5 and 3.5. At a pH of 4.5 and 5.5, the surface of the material was negatively charged, producing an electrostatic repulsive effect with HCr_2_O_7_^−^ and Cr_2_O_7_^2−^, which was unfavorable for adsorption. In addition, the decreased H^+^ in the solution was not favorable for the conversion of Cr(VI) to Cr(III). Under acidic conditions, the reduction reaction was more inclined to occur, while under neutral and alkaline conditions, Cr(III) existed as Cr(OH)_3_ on the surface of the Bi_2_O_3_ catalysts, inhibiting the photocatalytic activity of the photocatalysts [31].

**Figure 10:**
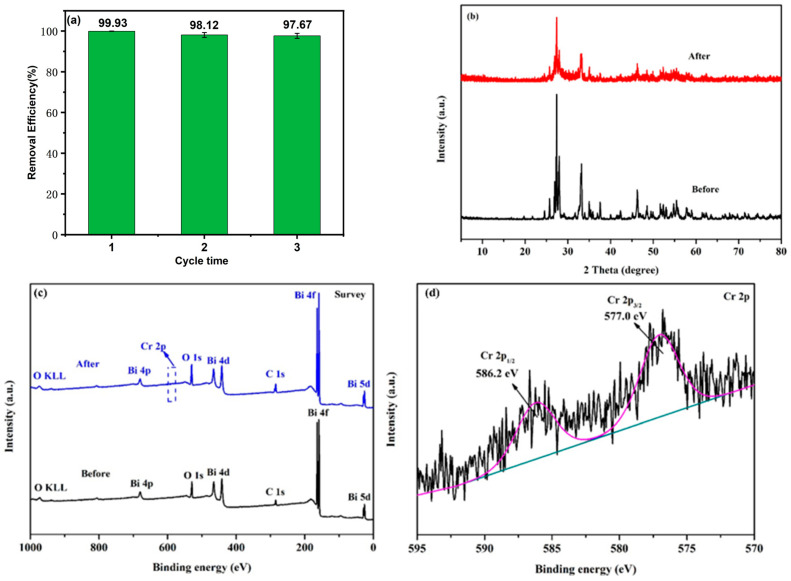
(**a**) Reusability of Bi_2_O_3_(DMF/MeOH-3)-400 in the photocatalytic reduction of Cr(VI). Comparison of (**b**) XRD patterns, (**c**) XPS survey before and after Bi_2_O_3_(DMF/MeOH-3)-400 was used. (**d**) Cr 2p spectra after Bi_2_O_3_(DMF/MeOH-3)-400 was used.

The reusability and stability of a photocatalyst are important properties that are necessary for expanding its practical application. To evaluate these properties for Bi_2_O_3_(DMF/MeOH-3)-400, three cycle experiments were performed. After each run, the catalyst was collected, washed several times with a 0.1 mmol·L^−1^ HNO_3_ solution and anhydrous ethanol, and then dried prior to the next run. As shown in Figure 10a, at a pH of 2.5, the Bi_2_O_3_(DMF/MeOH-3)-400 material was not significantly deactivated after three cycles and still achieved 99.10% Cr(VI) removal. In addition, as shown in Figure 10b,c, there was no significant change in the shape of the XRD or XPS spectra before and after the reaction, which indicated that the Bi_2_O_3_(DMF/MeOH-3)-400 material had good stability and reusability. Figure 10d shows the XPS spectrum of Cr 2p after the reaction. The peaks at 577.0 eV and 586.2 eV corresponded to Cr 2p_3/2_ and Cr 2p_5/2_ of Cr(III), respectively, which indicated that the Cr(VI) in the solution was reduced to Cr(III) by the Bi_2_O_3_(DMF/MeOH-3)-400 material [32].

Table 1 presents a comparison of the photocatalytic reduction activity of Cr(VI) by different photocatalysts. The Bi_2_O_3_(DMF/MeOH-3)-400 material prepared in this study showed certain advantages in terms of dosage, reaction time, and removal rate.

### 2.3. Possible Photocatalytic Mechanism

**Figure 11:**
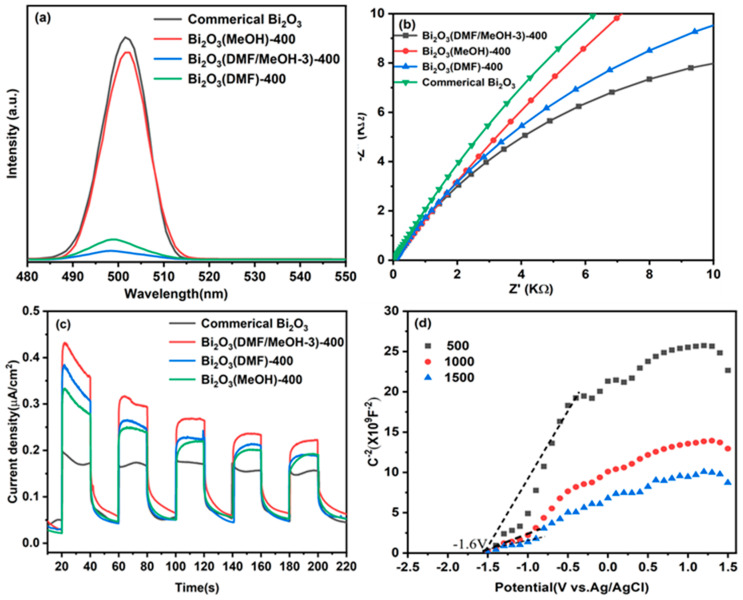
(**a**) Steady−state PL spectra, (**b**) EIS Nyquist plots, and (**c**) TPR plots of prepared Bi_2_O_3_ and commercial Bi_2_O_3_. (**d**) Mott−Schottky plots of prepared Bi_2_O_3_ at 500, 1000, 1500 Hz.

The photocatalytic activity of a catalyst mainly depends on the efficiency of electron–hole separation and electron migration [39]. The PL spectra of the Bi-BTC-derived Bi_2_O_3_ material and commercial Bi_2_O_3_ are shown in Figure 11a; they were excited at 440 nm with emission peaks in the 480–530 nm range. The PL signals of the Bi-BTC-derived Bi_2_O_3_ materials were all lower than those of commercial Bi_2_O_3_. Among them, Bi_2_O_3_(DMF/MeOH-3)-400 had the lowest PL intensity, which indicated a relatively low complexation rate of electrons and holes, implying more charge carriers in the reaction system [40]. To evaluate photogenerated e^−^–h^+^ separation, charge transfer, and the transient photocurrent response, electrochemical tests were performed [41]. The Nyquist plot of the Bi-BTC-derived Bi_2_O_3_ material and the commercial Bi_2_O_3_ (Figure 11b) indicates lower resistance or faster charge transfer by a smaller semicircle radius [34]. As shown in the figure, Bi_2_O_3_(DMF/MeOH-3)-400 had the smallest radius, confirming that it had the lowest charge transfer resistance and the highest e^−^–h^+^ separation efficiency. Equivalent circuits were fitted using an EIS test, where R1, R2, and C1 are the solution resistance, electron transfer resistance, and capacitive reactance due to diffusion effects in the system, respectively [42]. As shown in Figure 11c for the transient photocurrent response (TPR) curve, the Bi-BTC-derived Bi_2_O_3_ material had a stronger photocurrent density compared with the commercial Bi_2_O_3_ (0.2 μA/cm^2^), with the highest photocurrent density (0.4 μA/cm^2^) demonstrated by Bi_2_O_3_(DMF/MeOH-3)-400, confirming it to be the most efficient material for photogenerated carrier separation and transmission [43]. In addition, Mott–Schottky plots were used to investigate the flat-band potential (E_fb_) of the photocatalysts. As shown in Figure 11d, the curve of Bi_2_O_3_(DMF/MeOH-3)-400 had a positive slope, indicating that the Bi_2_O_3_ photocatalyst was an n-type semiconductor [35]. The E_fb_ of Bi_2_O_3_(DMF/MeOH-3)-400 was −1.60 V (vs. Ag/AgCl), which was transformed to a standard hydrogen electrode (E_NHE_) potential using the following equation [35]:

E_NHE_ = E_Ag/AgCl_ + 0.059 × pH + E°_Ag/AgCl_ (2)

where E°_Ag/AgCl_ is 0.197 V, and the pH was 6.0 under the test conditions.

The calculated E_fb_ for Bi_2_O_3_(DMF/MeOH-3)-400 was −1.05 V (vs. NHE). In addition, for many n-type semiconductors, the conduction band potential (E_CB_) is generally about 0.1 V more negative than the flat-band potential. Therefore, the E_CB_ of Bi_2_O_3_(DMF/MeOH-3)-400 was roughly estimated to be −1.15 eV. The valence band potential was obtained from the equation E_VB_ = E_CB_ + E_g_, and the E_VB_ of Bi_2_O_3_(DMF/MeOH-3)-400 was 1.69 eV.

**Figure 12:**
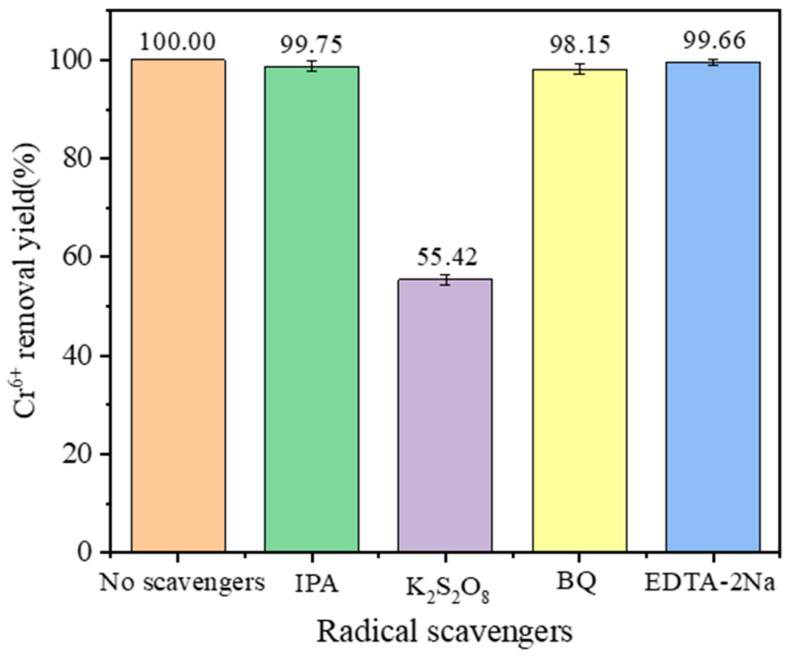
Reactive species trapping experiments of Bi_2_O_3_(DMF/MeOH-3)-400.

Reactive species capture experiments were conducted to elucidate the reaction mechanism. The active substance capture agents used in this study were EDTA-2Na (h^+^), isopropanol (IPA) (·OH), 1,4-benzoquinone (BQ) (·O_2_^−^), and K_2_S_2_O_8_ (e^−^). As shown in Figure 12, at a pH of 2.5, Cr(VI) removal was clearly inhibited by K_2_S_2_O_8_, due to the fact that K_2_S_2_O_8_ traps electrons, thus decreasing the number available for Cr(VI) reduction. In the presence of EDTA-2Na, the photocatalytic reduction efficiency of Cr(VI) remained high, which was attributed to the capture of h^+^ during the reduction of Cr(VI), which promoted the separation of photogenerated electrons and holes, thus allowing more electrons to participate in the reduction of Cr(VI). IPA and BQ had little effect on the photocatalytic reduction of Cr(VI). From this, we concluded that e^−^ was the main active substance in the photocatalytic reduction of Cr(VI) [44].

**Scheme 2:**
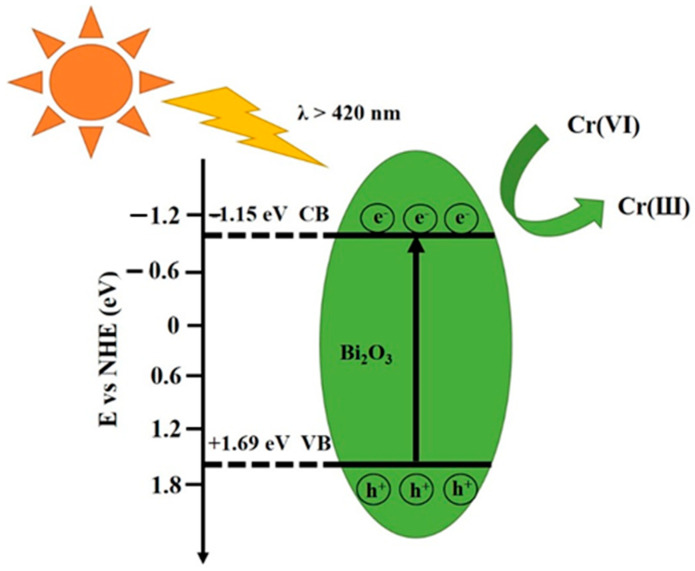
Proposed photocatalytic mechanism of Bi_2_O_3_(DMF/MeOH−3)-400.

Based on these results, a possible mechanism of Cr(VI) photocatalytic reduction over the Bi_2_O_3_(DMF/MeOH-3)-400 prepared in the present study was proposed, as depicted in Scheme 2. Under visible light irradiation, the electrons (e^−^) in the valence band (VB) of Bi_2_O_3_ are excited to the conduction band (CB). Cr(VI) is adsorbed on the surface of the material due to electrostatic adsorption after material protonation. Under visible light irradiation (λ > 420 nm), the Bi_2_O_3_(DMF/MeOH-3)-400 material is excited, the photogenerated electrons leap from VB to CB, and the e^−^ in CB participates in the conversion of Cr(VI) to Cr(III) as the main active substance. At a low pH, the main forms of chromium are HCrO_4_^−^ and Cr_2_O_7_^2−^, and the reaction equation of photocatalytic reduction of Cr(VI) is as follows: Cr_2_O_7_^2−^ + 14H^+^ + 6e^−^ → 2Cr_3_^+^ + 7H_2_O; under acidic conditions, reduction reactions are more likely to occur. As shown in this study, Bi-BTC was converted into rod-like Bi_2_O_3_ under high-temperature pyrolysis. Because the Bi ions were well distributed within the skeleton of the MOFs, the formed Bi_2_O_3_ was uniformly distributed within the rod-like structure. In other words, the rods were formed by the uniform arrangement of Bi_2_O_3_ nanoparticles. This multistage porous structure of hybrid systems can improve visible light absorption and hamper the recombination of photogenerated electron–hole pairs, thus increasing the number of electrons involved in the photoreduction reactions of Cr(VI) to Cr(III) for increased photoactivity.

## 3. Materials and Methods

### 3.1. Reagents and Materials

All chemicals were purchased from Adamas Reagent Co., Ltd. (Shanghai, China) and used without purification.

### 3.2. Fabrication of Bi-BTC Precursor and Bi-BTC-Derived Bi_2_O_3_ Materials

Bi-BTC was synthesized by referring to the previous literature with minor modifications [45]. Briefly, 150 mg of Bi(NO_3_)_3_·5H_2_O and 750 mg of H_3_BTC were dispersed in 60 mL of a solvent. The solvents were DMF, MeOH, DMF/MeOH-1 (1:1), DMF/MeOH-2 (2:1), DMF/MeOH-3 (3:1), and DMF/MeOH-4 (1:3). The mixture was stirred ultrasonically until dissolved, transferred to a 100 mL reactor, and placed in an oven at 120 °C to react for 24 h. After cooling, the white precipitate was collected by centrifugation and washed three times with DMF and MeOH. The washed sample was transferred to a vacuum drying oven at 60 °C and dried for 12 h to obtain a white powder. The samples were named Bi-BTC(DMF), Bi-BTC(MeOH), Bi-BTC(DMF/MeOH-1), Bi-BTC(DMF/MeOH-2), Bi-BTC(DMF/MeOH-3), and Bi-BTC(DMF/MeOH-4).

A certain amount of Bi-BTC was homogeneously dispersed in a crucible without a cover and calcined at 300 °C, 400 °C, and 500 °C. The temperature rate increase was 2 °C/min, and the calcination time was 2 h. The residue was cooled to room temperature to give a light yellow powdery material. The samples were named Bi_2_O_3_(DMF)-400, Bi_2_O_3_(MeOH)-400, Bi_2_O_3_(DMF/MeOH-1)-400, Bi_2_O_3_(DMF/MeOH-2)-400, Bi_2_O_3_(DMF/MeOH-3)-300, Bi_2_O_3_(DMF/MeOH-3)-400, Bi_2_O_3_(DMF/MeOH-3)-500, and Bi_2_O_3_(DMF/MeOH-4)-400.

### 3.3. Characterization Methods

The physicochemical properties of the materials were characterized through a series of techniques, including X-ray diffraction (XRD, TTR III, Rigaku Ltd., Tokyo, Japan), Brunauer–Emmett–Teller (BET, ASAP 2460, Micromeritics Instrument Co., Ltd., Shanghai, China), Scanning electron microscopy (SEM, Nova NanoSEM 450, FEI Corporation, Shanghai, China), Transmission electron microscopy (TEM, JEM-2100, Japan Electronics Co., Ltd., Tokyo, Japan), Fourier transform infrared spectroscopy (FTIR, Nicoletis 10, Thermo Nicolet Corporation, Madison, WI, USA), X-ray photoelectron spectroscopy (XPS, Thermo Fisher Scientific, Waltham, MA, USA), Zeta potential (NonaBrook ZetaPlus, Brookhaven Instruments, Shanghai, China), UV–vis diffuse reflection spectra (UV-Vis DRS, UV-2600, Shimadzu Corporation of Japan, Tokyo, Japan) and Photoluminescence (PL, F-4700, Hitachi Scientific Instruments Co., Ltd., Beijing, China). The impedance spectra and the Mott–Schottky plots were measured in a Na_2_SO_4_ solution (0.2 M, pH 7.0) on an electrochemical workstation (CHI660D, Shanghai Chenhua Instrument Co., Ltd., Shanghai, China).

### 3.4. Photocatalytic Activity Measurement

The photocatalytic activity of the catalysts was evaluated in a quartz tube photoreactor (Beijing Perfectlight Ltd., Beijing, China). In a typical experiment, 20 mg of the photocatalyst was added to 50 mL of a Cr(VI) solution (10.0 mg·L^−1^) in a 50 mL quartz tube. The pH value of the solution was adjusted by adding 0.10 mol·L^−1^ NaOH or 0.10 mol·L^−1^ HCl solution. After 2 h of dark reaction, 2.5 mL of the reaction suspension was collected and filtered through a 0.22 μm filter, and the diphenylcarbazide (DPC) (Adamas Reagent Co., Ltd., Shanghai, China) [46] colorimetric method was used to measure Cr(VI) to determine whether adsorption equilibrium was reached. When the Cr(VI) solution reached equilibrium, the circulating condensate of the light reaction system and the LED light source (power = 10 W, λ > 420 nm) were turned on, and at specific intervals of time, 2.5 mL of the reaction suspension was collected, filtered, and then tested for the concentration of hexavalent chromium using the DPC colorimetric method at 540 nm. The removal yield was calculated as follows:

Removal yield = (C_0_ − C_t_)/C_0_ × 100% (3)

where C_0_ is the initial concentration of the Cr(VI) solution (mg·L), and C_t_ is the concentration of Cr(VI) after t minutes of the reaction (mg·L).

When the photocatalytic reaction completed, the remaining catalyst in the suspension was collected by centrifugation. The centrifuged sample was then washed with a 0.1 mol/L dilute nitric acid solution, anhydrous ethanol, and deionized water by shaking. The material was dried overnight in a vacuum oven at 60 °C. The obtained material was used until three cycles were completed.

## 4. Conclusions

In summary, the Bi_2_O_3_ photocatalyst was obtained by in situ oxidation of metal Bi in a Bi-BTC skeleton through thermal decomposition of the precursor. Both the solvent and calcination temperature had clear influence on the structure and photocatalytic activity of the materials. The Bi_2_O_3_ samples exhibited the rod-like morphology of the Bi-BTC precursor prepared in a mixed solvent and formed a porous structure of closely arranged Bi_2_O_3_ nanoparticles. This unique structure enabled the material to have a narrow bandgap, strong spectral absorption, and numerous exposed active sites, which improved the photocatalytic activity of the catalyst. Our results showed that the Bi_2_O_3_(DMF/MeOH-3)-400 material exhibited excellent adsorption and photocatalytic reduction of Cr(VI). This was due to the rod-shaped porous structure of the material, which led to the fast separation of electron–hole pairs and efficient electron transfer in the MOF-derived materials. In addition, the Bi_2_O_3_(DMF/MeOH-3)-400 material showed excellent stability and reusability, indicating that it has broad application prospects in environmental and aquatic ecosystems. There is still a large gap between the application of the prepared materials and the actual industrial environment, which is also a topic that we will explore in depth in the future. This work provides a new reference for the preparation of high-performance photocatalysts that can effectively degrade pollutants.

## Figures and Tables

**Table 1:** Comparison of the performance of different photocatalysts for the photocatalytic reduction of Cr(VI).

Photocatalyst	Light Source	pH	Dosage (g·L^−1^)	Concentration (mg·L^−1^)	Time (min)	Removal Rate (%)	Ref.
ZnFe_2_O_4_/CdS	1 kW Xe lamp (λ > 420 nm)	2.5	0.5	35	120	90	[33]
BiVO_4_/FeVO4@rGO	1 kW Xe lamp (λ > 420 nm)	2.5	0.6	20	90	90.9	[34]
Ag/AgCl-S@g-C_3_N_4_	300 W Xe lamp (λ > 420 nm)	2.5	0.1	10	60	60	[35]
Bi_4_O_5_Br_2_/Bi_2_S_3_	500 W Xe lamp	2.5	0.4	20	80	91.3	[36]
Fe_3_O_4_@ZIF-8@BiOCl	300 W Xe lamp (λ > 420 nm)	2.5	0.75	5	140	68	[37]
nylon-6@UiO-66-NH_2_	300 W Xe lamp (λ > 420 nm)	2.5	0.5	10	180	97	[38]
Bi_2_O_3_(DMF/MeOH-3)-400	10 W LED	2.5	0.4	10	90	100	In this paper

## Data Availability

Data available on request.

## Supplementary Materials

The following supporting information can be downloaded at https://www.mdpi.com/article/10.3390/ijms252313052/s1.
