# Supplementary material for: A Rod-like Bi2O3 Photocatalyst Derived from Bi-Based MOFs for the Efficient Adsorption and Catalytic Reduction of Cr(VI)
Source: Int J Mol Sci. 2024 Dec 4;25(23):13052. doi: 10.3390/ijms252313052 (PMC11641822; doi:10.3390/ijms252313052)
Supplement: Supplementary file 1 [file ijms-25-13052-s001.zip › ijms-3273362-supplementary.pdf]

Supporting Information

A Rod-like Bi_2O_3 Photocatalyst Derived from Bi-Based MOFs for the Efficient Adsorption and Catalytic Reduction of Cr(VI)

Qin Fang [1,2], Luying Chen [1,2], Qiucheng Fu [1,2], Yongjuan Chen [1,2], Jiao He [1,2], Liang Jiang [2,3], Zhiying Yan[1,2,*], Jiaqiang Wang[2,4,*]

1 School of Chemical Sciences & Technology, Yunnan University, Kunming 650091, China

2 Yunnan Province Engineering Research Center of Photocatalytic Treatment of Industrial Wastewater, Yunnan University, Kunming 650091, China

3 Engineering Institute, Yunnan University, Kunming 650091, China

4 School of Materials & Energy, Yunnan University, Kunming 650091, China

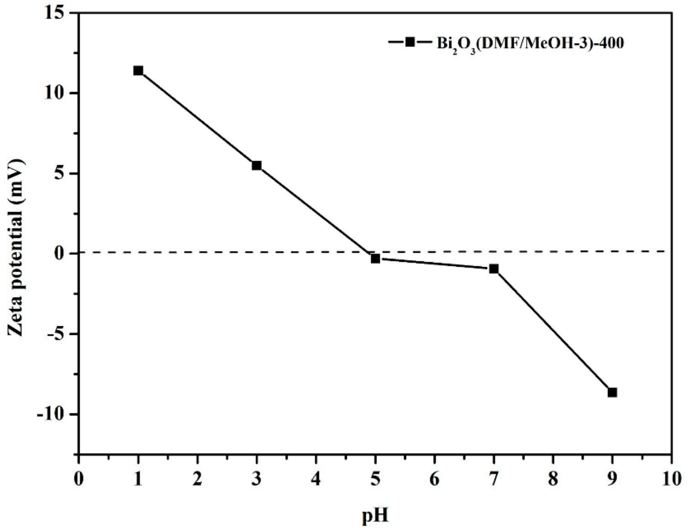

Figure. S1. Variation of the Zeta potential of Bi_2O_3(DMF/MeOH-3)-400 suspended in water as a function of pH.

Table S1. Specific surface area and pore structure data of the samples.

Samples	S_{BET} (m^2/g)	Pore volume (cm^3/g)	Pore size (nm)
Bi-BTC(DMF)	5.53	0.013	24.36
Bi-BTC(MeOH)	3.30	0.006	18.65
Bi-BTC(DMF/MeOH-1)	15.69	0.017	28.80
Bi-BTC(DMF/MeOH-2)	17.58	0.026	22.59
Bi-BTC(DMF/MeOH-3)	17.93	0.033	23.86
Bi-BTC(DMF/MeOH-4)	8.98	0.013	14.40
Bi_2O_3(DMF)-400	2.83	0.005	36.79
Bi_2O_3(MeOH)-400	2.27	0.004	34.82
Bi_2O_3(DMF/MeOH-1)-400	2.69	0.007	31.86
Bi_2O_3(DMF/MeOH-2)-400	3.13	0.008	17.48
Bi_2O_3(DMF/MeOH-3)-400	6.60	0.015	23.66
Bi_2O_3(DMF/MeOH-4)-400	3.22	0.007	18.08

Table S2. The band gap and photocatalytic activities of as-prepared samples.

Samples	E_g (eV)
Bi_2O_3(DMF)-400	2.85
Bi_2O_3(MeOH)-400	2.88
Bi_2O_3(DMF/MeOH-3)-400	2.84
Commercial Bi_2O_3	2.89

Table S3. Apparent rate constant of as-prepared samples and commercial Bi_2O_3.

Samples	k(min^{-1})
Bi_2O_3(DMF)-400	0.009
Bi_2O_3(MeOH)-400	0.005
Bi_2O_3(DMF/MeOH-1)-400	0.016
Bi_2O_3(DMF/MeOH-2)-400	0.021
Bi_2O_3(DMF/MeOH-3)-400	0.035
Bi_2O_3(DMF/MeOH-4)-400	0.015
Commercial Bi_2O_3	0.005

Table S4. Apparent rate constant of photocatalytic reduction of Cr(VI) with different initial pH.

Initial pH of Cr(VI)	2.5	3.5	4.5	5.5
k(min^{-1})	0.035	0.011	0.001	0.0005

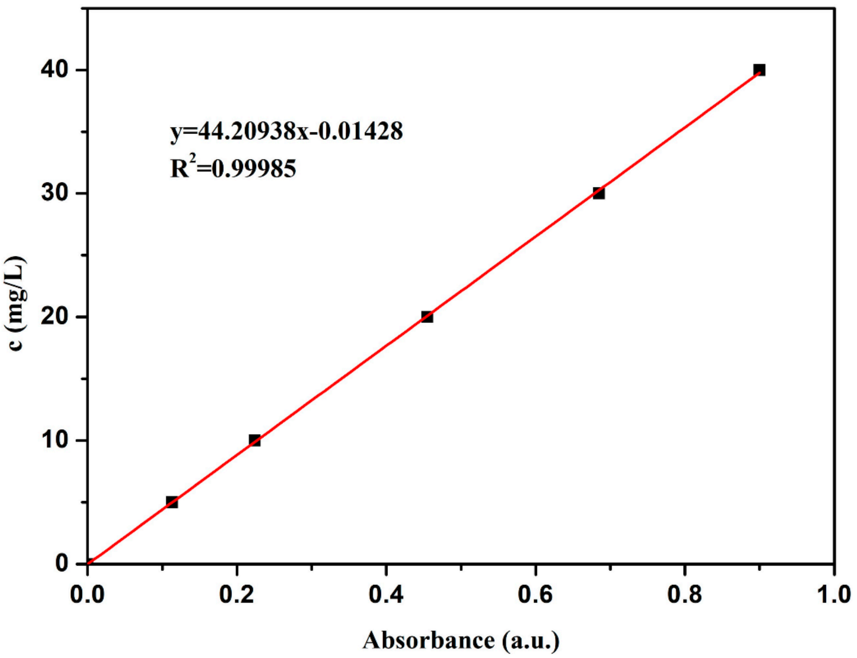

Figure. S2. Standard curve of Cr(Ⅵ).

The determination of hexavalent chromium in the experiment refers to GB 746-87 diphenylcarbazide (DPC) colorimetric method for the determination of hexavalent chromium. The specific determination method is to weigh 0.20 g of diphenylcarbazide dissolved in 50 mL of acetone solution as a chromogenic agent, prepare a solution of sulfuric acid (1:1) and phosphoric acid (1:1), (1:1) is the volume ratio of concentrated sulfuric acid or phosphoric acid to water. During each measurement, take 400 μL of the solution to be tested in a 10 mL colorimetric tube, add 400 μL of chromogenic agent, add four drops of sulfuric acid (1:1) and phosphoric acid (1:1) solution dropwise, add deionized water and dilute it to 10 mL of marking, shake well, let stand for 10 min for color development, and measure the absorbance of the solution at 540 nm with an ultraviolet-visible spectrophotometer.

To draw the hexavalent chromium standard curve, weigh 2.83 g of $K_2Cr_2O_4$ crystals, add deionized water to sonically dissolve and transfer to a 1000 mL volumetric flask for bandwidth evaluation to obtain a 1000 ppm Cr (VI) solution. Measure 5 mL, 10 mL, 20 mL, 30 mL, and 40 mL of the above 1000 ppm hexavalent chromium solution with a measuring cylinder, pour it into a 1000 mL volumetric flask, bandwidth evaluation shake well, and configure to obtain a standard Cr (VI) solution of 5 ppm, 10 ppm, 20 ppm, 30 ppm, and 40 ppm. The diphenylcarbazide (DPC) colorimetric method was used for determination, and the method could maintain good linearity at 0.0mg/L-40mg/L under experimental conditions, and the linear equation was obtained as Y = 44.20938X-0.01428 (R_2 = 0.99985), Y is the concentration mg/L, X is the absorbance.
